# Integration of Developed Mathematical Model for Predicting and Monitoring the Spread of Epidemics and Pandemics: The Case of COVID‐19 in Rwanda

**DOI:** 10.1002/puh2.70287

**Published:** 2026-06-01

**Authors:** Jean Marie Ntaganda, Innocent Ngaruye, Denis Ndanguza

**Affiliations:** ^1^ Department of Mathematics School of Science College of Science and Technology University of Rwanda Kigali Rwanda

**Keywords:** COVID‐19, dashboard, mathematical model

## Abstract

This study presents a novel, context‐specific mathematical modeling framework for predicting and monitoring COVID‐19 transmission in Rwanda, addressing the increasing demand for integrated and data‐driven public health decision‐support tools. The model incorporates localized epidemiological dynamics, transmission pathways, and major intervention measures, including lockdowns, vaccination, quarantine, and home‐based care, which is considered a critical factor influencing disease transmission. Using a system of differential equations, the framework models transition among infected, home‐based care, hospitalized, recovered, and deceased populations. The model was calibrated and validated using Rwanda's national COVID‐19 surveillance data collected between March 2020 and December 2022, covering multiple epidemic waves and intervention phases. Calibration utilized time‐series data on confirmed cases, active infections, hospitalizations, intensive care unit admissions, recoveries, and mortality. Detailed home‐based care records improved the estimation of transmission and recovery parameters. Parameter optimization was performed through least‐squares fitting and cross‐validation techniques. Model performance was assessed using the root mean square error (RMSE) and the coefficient of determination (*R* ^2^) metrics. Validation through out‐of‐sample predictions demonstrated strong accuracy in capturing infection trends and healthcare burden. An interactive dashboard complements the framework by enabling real‐time analytics, forecasting, and evidence‐based public health decision‐making.

## Graphical abstract

A context‐specific mathematical modeling framework is developed to predict and monitor COVID‐19 transmission in Rwanda using a system of differential equations. The model incorporates localized epidemiological dynamics and key interventions, including lockdowns, vaccination, quarantine, and home‐based care, to represent transitions among infected, hospitalized, recovered, and deceased populations. It is calibrated and validated using national surveillance data from 2020 to 2022 across multiple epidemic waves. Performance is evaluated using root mean square error (RMSE) and coefficient of determination (R2), demonstrating strong predictive accuracy. An interactive dashboard supports real‐time analytics, forecasting, and evidence‐based public health decision‐making.

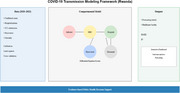

## 1 Problem Statement

Emerging and re‐emerging epidemics and pandemics of infectious diseases continue to be a serious public health concern in Rwanda and worldwide [1, 2]. Cognisant of the progress made by the government of Rwanda in controlling and managing the spread of epidemic diseases, the role of science and innovation has been and remains important in informing and generating the evidence needed for policy actions [3]. The most recent experience is the positive impacts of science and innovation in reducing the spread and impacts of the COVID‐19 pandemic. During the COVID‐19 outbreak, 231 countries have been affected by the pandemic, and various mathematical models describing the dynamics of COVID‐19 were developed for other countries [4] but not for Rwanda. Consequently, Rwanda had to rely on those models developed without necessarily considering its specific context and characteristics. Before this model was developed, policymakers struggled to predict the number of people who would be infected. However, this model now enables them to anticipate how many individuals will be affected by COVID‐19. As a result, this research has been conducted to address this challenge by developing a mathematical model capable of predicting and monitoring pandemic diseases.

## 2 Methods, Approach, and Key Findings

A compartmental transmission model was developed to study the dynamics of COVID‐19 [5]. This model consisted of 10 compartments: susceptible (*S*), exposed (*E*), quarantine (*Q*), infectious (*I*), undocumented infectious (*I_u_*), home‐based‐care (*I_h_*), hospitalized (*H*), vaccinated (*V*), recovered (*R*), and dead (*D*). It was developed specifically for the Rwandan context using a transfer diagram, which connects various compartments through different parameters. Home‐based care was included as a distinct variable to reflect local practices. Some parameters were sourced from existing literature, whereas others were estimated using available data by the least squares method. The model is defined as

$$\begin{array}{c} \frac{dS}{dt}=\lambda +\sigma Q-\left(\frac{\beta_{E}E+\beta_{I_{u}}I_{u}+\beta_{I}I+\beta_{I_{h}}I_{h}+\beta_{H}H}{N}+\varepsilon +D_{s}\right)S+\left(1-\mu \right)V\\ \frac{dE}{dt}=\frac{\beta_{E}E+\beta_{I_{u}}I_{u}+\beta_{I}I+\beta_{I_{h}}I_{h}+\beta_{H}H}{N}S-\alpha E\\ \frac{dI}{dt}=\alpha E+\delta Q-\omega_{1}I\\ \frac{dQ}{dt}=\Lambda +\varepsilon S-\left(\delta +\sigma \right)Q\\ \frac{d_{I_{u}}}{dt}=T_{1}\omega_{1}I-\omega_{2}I_{u}\\ \frac{d_{I_{h}}}{dt}=T_{2}\omega_{1}I-\omega_{3}I_{h}\\ \frac{dH}{dt}=\left(1-T_{1}-T_{2}\right)\omega_{1}I+\left(1-\theta \right)\omega_{3}I_{h}-\omega_{4}H\\ \frac{dR}{dt}=\left(1-\gamma \right)\omega_{2}I_{u}+\eta \omega_{4}H+\theta \omega_{3}I_{h}-D_{R}R\\ \frac{dD}{dt}=\gamma \omega_{2}I_{u}+\left(1-\eta \right)\omega_{4}H\\ \frac{dV}{dt}=D_{s}S+D_{R}R-\left(1-\mu \right)V. \end{array}$$

The model assumes a closed population with homogeneous mixing and constant demographic conditions over the study period. Disease transmission follows compartmental transitions governed by defined rates. Key parameters include the transmission rate, quarantine rate (*ε*), infection progression rate (*α*), and recovery rates (*ν*, *θ*, *γ*) across different compartments. The proportions of undocumented infections (*τ*_1_) and home‐based‐care cases (*τ*_2_) are explicitly included. Vaccination is modeled through daily rates (*D_s_*, *D_r_*) and efficacy (*μ*), with allowance for waning immunity. Parameters are derived from literature and estimated via nonlinear least‐squares fitting, with uncertainty assessed using bootstrap resampling and sensitivity analyses to ensure robustness.

The transition rate from susceptible to infectious is defined, whereas *ε* represents the rate at which susceptible individuals enter quarantine. Within quarantine, *σ* denotes the rate at which individuals remain uninfected, and *δ* represents the rate at which quarantined individuals become infected. The fractions of infected individuals who are undocumented (*τ*_1_) or monitored at home (*τ*_2_) are also specified. The model assumes a mean latent period of 1/*α* days, so *α* is the rate at which exposed individuals progress to being infectious. Average durations of infection are 1/*ν*, 1/*θ*, and 1/*γ* days for individuals in the *H*, *I_h_*, and *I_u_* compartments, respectively, with transition rates between infectious compartments represented by *w*_1_, *w*_2_, *w*_3_, and *w*_4_. Vaccination is incorporated gradually, with daily fractions *D_s_* and *D_r_* of susceptible and recovered individuals receiving vaccines, respectively, and an assumed vaccine efficacy of *μ*. We assumed that vaccinated individuals gain partial or full immunity, reduced infection risk, and possible waning immunity over time, allowing transition back to susceptible or lower protection compartments.

This study introduces a context‐specific COVID‐19 transmission model tailored to Rwanda, distinguishing it from existing approaches developed for other settings. Its novelty lies in integrating a home‐based‐care compartment that reflects local clinical practices, alongside explicit representation of undocumented infections, which significantly influence transmission dynamics. The model incorporates real‐time calibration with uncertainty quantification using bootstrap methods, enabling reliable short‐term forecasting without large‐scale data requirements. Furthermore, it is coupled with an interactive dashboard supporting real‐time monitoring and decision‐making. The provision of implementations in MATLAB enhances reproducibility, accessibility, and practical adoption for epidemic preparedness and response in resource‐constrained settings.

**FIGURE 1:**
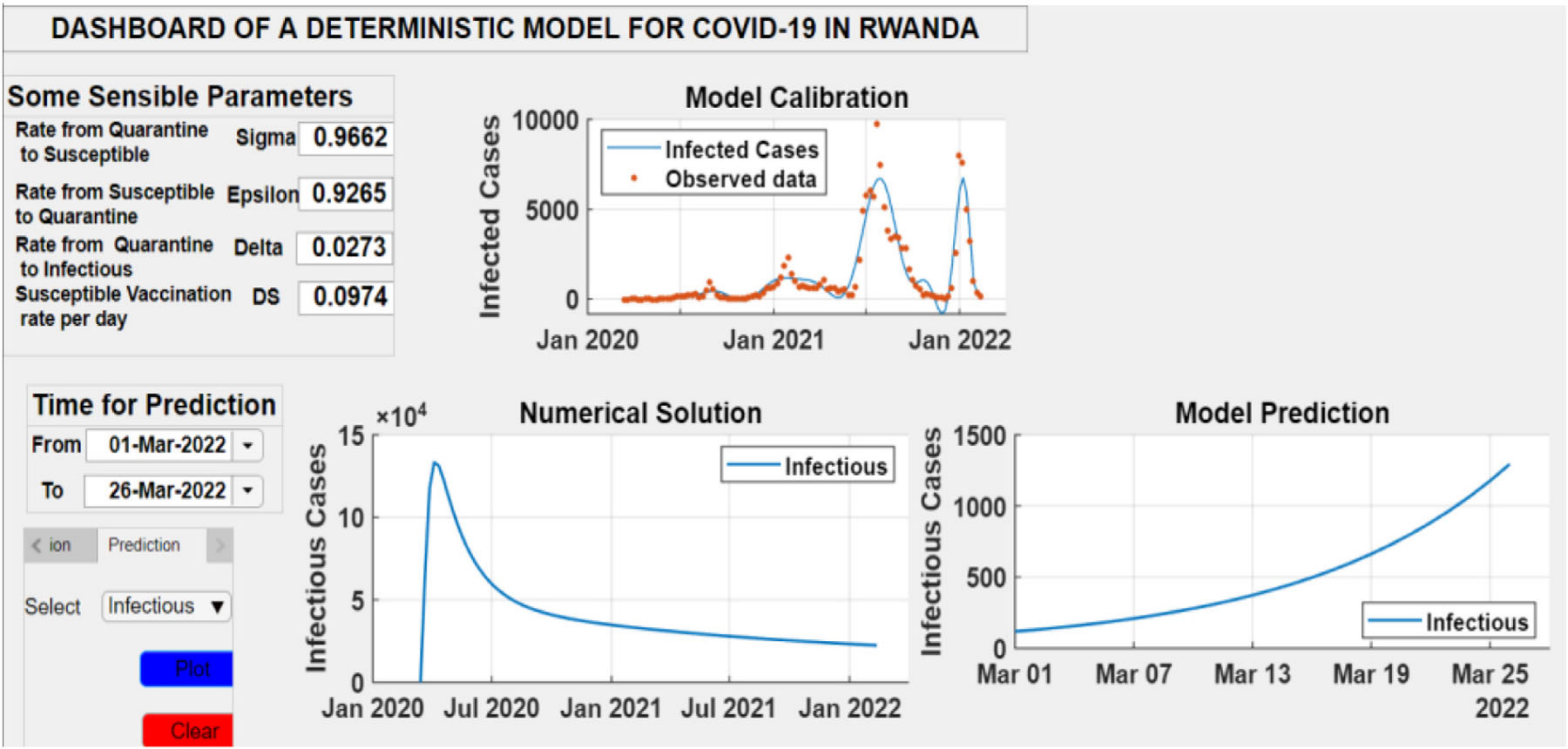
The designed MATLAB dashboard for data fitting, computing solutions, and predicting the spread of COVID‐19. It indicates that we need to set appropriate parameters for the model in the top left corner, and below that, we must specify the starting and ending times for the prediction process. After selecting the model variable from the drop‐down menu and clicking the push button for plotting, the calibration is displayed in the top right, whereas the solution and predicted variation are shown in the bottom right, respectively.

The model was analyzed and validated to ensure its accuracy in predicting and monitoring the spread of COVID‐19, without relying on large‐scale data as in Nuha et al. [6]. Parameter values were estimated using nonlinear least‐squares calibration against observed daily case data. To quantify uncertainty, a bootstrap resampling procedure with 1000 replicates was employed to generate 95% credible intervals for all calibrated parameters and model trajectories. Robustness was further evaluated by assessing the model under alternative prior assumptions and conducting sensitivity analyses on reporting delays (ranging from 1 to 7 days) and under‐ascertainment multipliers. Consequently, the projections depicted in Figure 1 represent parameter estimates that remained consistent across these uncertainty and sensitivity scenarios.

The findings revealed that 64.74% of all infected individuals were unreported. None of the existing models accurately represent the dynamics of the disease in the Rwandan context, making it impossible to import other models. This unreported group served as a significant contributor to the transmission of the pandemic within the Rwandan population.

To evaluate the enhanced accuracy and efficacy of our proposed model, we conducted simulations to compare key outcome variables, such as mortality and transmission rates, with those from an established compartmental mathematical model introduced by Serovajsky [7]. The comparison revealed that the proposed mathematical model, which incorporates a home‐based‐care compartment, led to an 18.21% reduction in the transition rate from the infectious to hospitalized compartments. Additionally, there was a slight decrease in the number of hospitalized patients who succumbed to the disease. These findings highlight the effectiveness of the integrated home‐based‐care compartment into the mathematical modeling framework for COVID‐19.

A real‐time dashboard was developed to visually display essential metrics, continuously updated with the most recent data. This allows public health officials and policymakers to track the disease's progression and the effectiveness of interventions as circumstances change.

The dashboard has been designed to utilize real‐time data sources, enhancing the model's impact, usability, and accessibility. Although other tools presented in HA et al. [8] can be used, the model provides executable files for MATLAB software and offers robust decision‐support capabilities, including prediction, real‐time visualization, and user‐friendly interaction. In addition, the model is implemented in Python or R, ensuring accessibility, reproducibility, and broader adoption beyond MATLAB for epidemic modeling. Using Python or R ensures open‐source code availability, detailed documentation, improved accessibility, reproducibility, and broader adoption beyond MATLAB for epidemic modeling. A sample screenshot of the dashboard is presented in Figure 1, whereas the predicted model variables are shown in Table 1. The dashboard also presents prediction intervals derived from the calibrated parameter distributions, enabling users to observe 95% credible bands surrounding each projection. This enhancement ensures that predictions are accompanied by their uncertainty, thereby enhancing the model's utility for decision‐making.

**TABLE 1:** Reported number as of February 28, 2022, compared to the predicted numbers for some model variables.

Variable	Reported number	Predicted number (next 6 weeks)
Infected	129,502	133,229
Infected but undocumented	NA	28,000
Infected and receiving home‐based care	NA	16,000
Hospitalized	130,957	100,000
Deaths	1457	1358
Exposed	NA	0
Quarantined	NA	0
Vaccinated	8,815,573	8,653,110
Recovered	132,039	290,000

The study acknowledges limitations, including behavioral fatigue affecting adherence to control measures, evolving booster dose strategies, and changing vaccine effectiveness. Data constraints, parameter uncertainty, and unmodeled social heterogeneity may influence predictions. These factors highlight the need for cautious interpretation and continuous model updates to maintain accuracy and policy relevance.

## 3 Conclusions

Integrating the developed mathematical models into Rwanda's epidemic response framework represents a crucial advancement in the country's capacity to predict, monitor, and control the spread of infectious diseases. This initiative builds on the successes demonstrated during the COVID‐19 pandemic and positions Rwanda to address future public health challenges with a proactive, data‐driven approach. By continuing to use and refine these models, Rwanda will strengthen its ability to safeguard public health and bolster the resilience of its healthcare system, ensuring preparedness for future epidemics. The developed dashboard has shown the accuracy in predicting the spread of COVID‐19, and it can be customized to other pandemic diseases. With the dashboard, decision‐makers can implement quick strategies to mitigate the spread of the pandemic basing themselves on facts.

### 3.1 Future Work

As demonstrated in LaJoie et al. [9], future research should integrate time‐series data on public compliance, detailed vaccine rollout and booster coverage, and models capturing behavioral fatigue or the decay of policy adherence over time. The next orientation is also to propose a hybrid compartmental–deep learning COVID‐19 model enabling adaptive parameter estimation, with a flexible framework supporting future integration of ensemble methods and optimization algorithms for robust, dynamic tuning [10]. The subsequent studies could implement cross‐validation over multiple time periods and locations, conduct sensitivity analyses, and assess model performance using root mean square error (RMSE), mean absolute error (MAE), and Akaike information criterion (AIC) metrics [11].

### 3.2 Recommendations

The following recommendations should be considered for the prediction and monitoring of epidemic diseases:

- Government policies related to health should consider including the proposed mathematical models that account for the specific conditions affecting the spread of a particular epidemic disease, while also considering Rwanda's context in disease monitoring. When identifying infected cases that could contribute to the transmission of epidemic infectious diseases, it is important to consider a specific group of unreported cases to mitigate the spread of such diseases
- The University of Rwanda and other partners may provide a platform where the dashboard of the proposed mathematical model can be published and accessed by any user who needs to use it for predicting and monitoring the spread of epidemic diseases in Rwanda.
- To ensure the long‐term operational use of the model, a comprehensive plan for model monitoring and refinement is essential. This plan includes scheduled recalibration of model parameters on a weekly or bi‐weekly basis, depending on the availability of data. Additionally, establish a human‐in‐the‐loop review mechanism where public health analysts validate model outputs and flag any anomalies. Furthermore, incorporate explicit communication of uncertainty through prediction intervals and scenario ranges displayed on the dashboard. These measures ensure transparency, continuous accuracy, and responsible communication of uncertainty to policymakers.

## Author Contributions

All authors contributed equally to the study conception, methodology, data analysis, manuscript preparation, revision, and approval of the final submitted version for publication consideration.

## Ethics Statement

This study received ethics clearance from the Institutional Review Board (IRB) of the College of Medicine and Health Sciences (CMHS) at the University of Rwanda for conducting research on COVID‐19 transmission in Rwanda [5].

## Conflicts of Interest

The authors declare no conflicts of interest.

## Data Availability

Data sharing is not applicable to this article as no datasets were generated or analyzed during the current study.
